# Guided Endodontic Management of Orthodontic‐Induced Canal Calcification Using Static Guide: A Case Report

**DOI:** 10.1155/crid/4788874

**Published:** 2026-08-13

**Authors:** Ihtisam Alam, Aravind R. Kudva, S. Vidhyadhara Shetty, Aboobacker Sidheeque

**Affiliations:** ^1^ Department of Conservative Dentistry and Endodontics, Yenepoya (Deemed to Be University) Dental College, Mangaluru, Karnataka, India

**Keywords:** canal obliteration, case report, CBCT, guided endodontics

## Abstract

**Background:**

Orthodontic treatment may induce pulpal calcific changes, including pulp stone formation, fibrotic tissue changes, and reduction in pulp volume, which may contribute to canal narrowing and, in some cases, canal obliteration. But with the introduction of guided endodontics, treating calcified tooth has become less complex and also helps in preservation of pericervical dentin when compared with traditional access cavity preparation.

**Case Report:**

The patient reported pain and sensitivity on both of the maxillary central incisors. Upon examination, the right maxillary central incisor had pulpal exposure requiring immediate treatment, whereas the left maxillary central incisor had a completely obliterated canal and showed no response to thermal and EPT. So, it was decided to treat 21 using static guide. The treatment was completed in two visits for Tooth 21 with very minimal discomfort. To close the midline diastema, a full coverage crown was placed.

**Conclusion:**

This case demonstrates the successful management of obliteration arising from orthodontic tooth movement using a static guide navigation system. The use of a static guide not only helped in preservation of pericervical dentin but also was less time‐consuming, hence causing minimal discomfort to the patient.

## 1. Introduction

The foundation of orthodontic treatment is the application of stresses to teeth, which may result in molecular changes to the periodontium′s tissue cells. [1] Aspects of the pulpal reaction to orthodontic therapy that could harm the tooth pulp include cell death, inflammation, and wound healing. According to published histology research, pulp reacts to orthodontically applied forces in a variety of ways, from necrosis to circulatory vascular stasis.

Inflammation‐associated mediators are released and accumulate when the pulp tissue is subjected to an orthodontically produced force. Pulpal responses following orthodontic forces encompass a spectrum of calcific changes, including diffuse or localized calcifications within the pulp space and partial or complete canal obliteration. These changes may occur after orthodontic treatment, particularly in teeth with prior trauma, but the relationship is not strictly causal and varies by individual biology and treatment parameters. Although some histologic studies suggest dentin bridge–like or tertiary dentin–like deposition, the predominant mechanism in obliteration is thought to involve altered pulpal cell activity and hard‐tissue deposition rather than a single uniform pathway [2].

It can be due to the reparative or resorptive response of odontoblasts and related cells [3], thereby, causing reduction of pulp space volume and leading to its complete obliteration.

When conventional approaches are used in clinical situations such as pulp canal calcification and dens evaginatus, it increases the risk of perforation and excessive tooth structure removal thereby compromising the treatment outcome. Although microscopes and cone‐beam computed tomography (CBCT) can provide guidance, it can be challenging for operators to perform the therapy manually without errors. With the use of minimally invasive endodontic access opening preparation approaches, this can be prevented. Guided endodontics (GE) uses computer‐aided navigation systems to aid in accurately locating and treating root canals.

A virtually planned and guided minimally invasive access cavity could help to preserve tooth structure and avoid perforations, which could lead to an improved long‐term prognosis, especially for teeth with calcified root canals, as utilizing guided access has demonstrated encouraging results. Additionally, the primary objective of endodontic, restorative, and modern dentistry techniques is to preserve tooth structure [4].

So, this case report presents the use of a static guide navigation system to locate an obliterated canal caused by excessive orthodontic force while preserving the tooth structure.

## 2. Case Presentation

**Figure 1:**
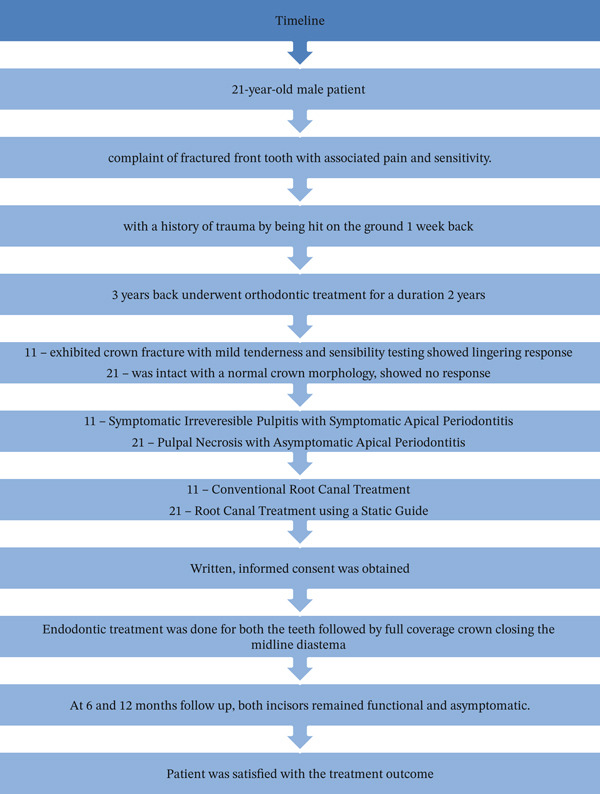
Timeline according to CARE reporting.

(Figure 1) A 21‐year‐old male patient reported with the complaint of fractured front tooth [5] with associated pain and sensitivity. The patient gave a history of trauma by being hit on the ground 1 week back which resulted in the fracture of Tooth 11 and also complained of sensitivity in Tooth 21. The medical history was noncontributory. The dental history revealed that 3 years back, the patient underwent fixed orthodontic treatment for a duration of 2 years.

**Figure 2:**
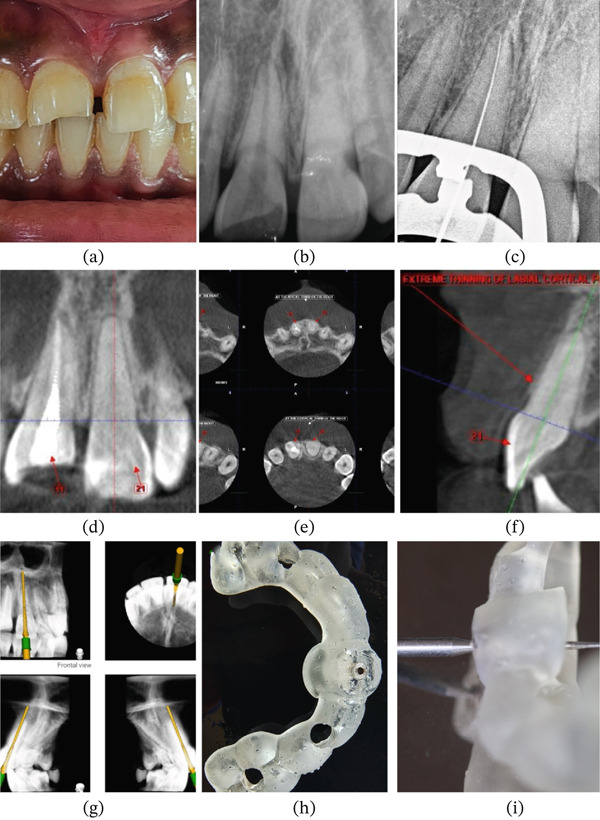
(a) Preoperative clinical image, (b) preoperative radiograph, (c) endodontic treatment of Tooth 11, (d–f) CBCT images, (g) planning of drill path using implant planning software, (h) fabricated endoguide, and (i) drill.

Tooth 11 exhibited a crown fracture involving the incisal third extending into dentin with a pinpoint pulpal exposure and mild tenderness on percussion. Tooth 21 was intact, with a normal crown morphology and no discoloration (Figure 2a). Probing depths around both teeth were within normal limits, and there were no sinus tracts. Thermal and electric pulp testing elicited an exaggerated lingering response in Tooth 11, whereas Tooth 21 showed no response to either cold or electric tests on repeated assessment. A timeline outlining the key components of case management is presented in the flowchart below.

A periapical radiograph of Tooth 11 demonstrated a normal root canal space and intact periapical bone, with radiolucency involving pulp. And for 21, complete obliteration of the root canal space from the coronal to apical third was noted (Figure 2b). The periodontal ligament (PDL) space and lamina dura appeared intact. Diagnosis for Tooth 11 was symptomatic irreveresible pulpitis with symptomatic apical periodontitis. Given the importance of the situation and complaint of the patient, 11 was endodontically treated (Figure 2c), and the patient was sent for CBCT imaging for Tooth 21 to better visualize the canal trajectory and the relationship of the root to the periapical region.

The CBCT scans confirmed complete obliteration of the canal in the cervical and middle thirds, with only a faint canal remnant apically. There was no periapical radiolucency, but the labial cortical plate overlying the root of Tooth 21 was extremely thin, particularly in the midroot region. Assessment of cross‐sectional slices indicated that any labially deviated access could lead to labial root perforation and cortical plate fenestration (Figure 2d–f).

Based on the subjective symptoms, negative response to sensibility tests, extreme thinning of the labial cortical plate, diagnosis for Tooth 21 was pulpal necrosis with asymptomatic apical periodontitis. Though periapical radiolucency was not visible in 2D radiographic image, CBCT revealed a thin labial cortical plate with canal obliteration indicating a high risk of future lesion development, the patient was given RCT as treatment option either conventionally or using a static guide. Patient agreed to continue treatment using a static guide as it will minimize removal of pericervical dentin which in turn reduce the risk of fracture and perforation.

An intraoral digital impression was obtained using an optical scanner, generating an STL file. Both DICOM data and STL file were exported and imported into implant planning software. Using the merged datasets, a virtual access path was planned from the incisal edge of Tooth 21 along the predicted canal trajectory, with the drill axis centered within the root (Figure 2g). A metallic sleeve position was designed within a tooth‐supported guide extending over the maxillary central incisors and adjacent teeth to maximize stability.

The static guide was fabricated using a stereolithographic 3D printer and biocompatible resin (Figure 2h,i). Sleeve of inner diameter bigger than the drill and a height of 7 mm was incorporated into the guide to prevent deviation of the drill from the original path. On the day of treatment for Tooth 21, fit of the guide was verified intraorally to ensure complete seating and stability, as improper seating is a known risk factor for guided drilling deviation and potential perforation.

**Figure 3:**
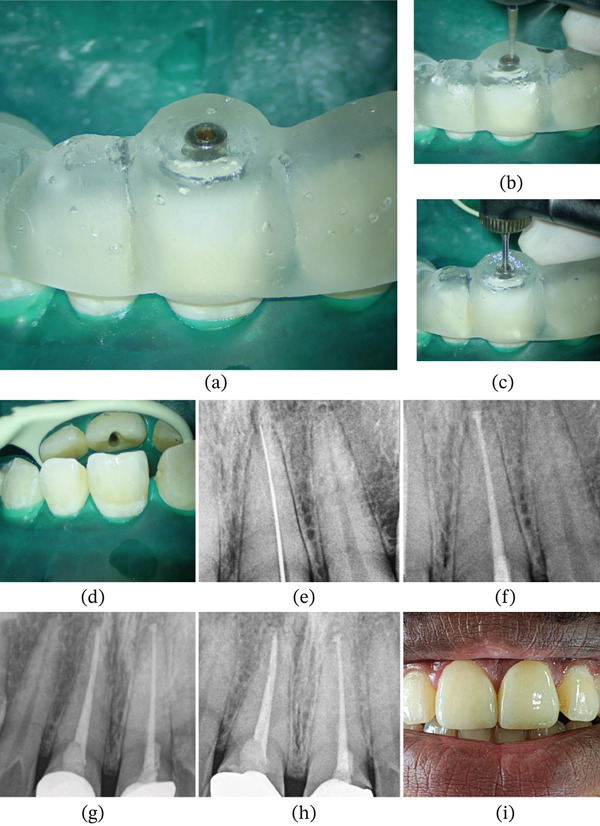
(a) Checking stability of the endoguide, (b) removal of enamel using high speed handpiece, (c) use of EndoGuide bur along the planned path, (d) conservative access achieved, (e) working length determined, (f) immediate postobturation radiograph, (g) 6‐month follow‐up radiograph, and (h–i) 12‐month follow‐up radiograph and clinical image.

The tooth was anesthetized using local anesthesia (Indoco Warren Lignox Lignocaine 2%, Indoco Remedies Ltd., India) and isolated with a rubber dam. The static guide was seated, and its stability was rechecked (Figure 3a). A long‐shank tapered bur compatible with the metallic sleeve was used at high speed to remove the enamel (Figure 3b). Then, the EndoGuide bur was used through the sleeve along the planned drill path (Figure 3c).

After reaching the planned depth, glide finder files (Mani, Japan) were used to gently negotiate the remaining part of the canal, and a glide path was established (Figure 3d). Working length was confirmed with an electronic apex locator and a radiograph (Figure 3e). The canal was instrumented using a VDW rotate blue system up to 35 4%, with irrigation using continuous chelation method. And in the second appointment, it was obturated using single cone of gutta‐percha and bioceramic sealer (Meta CeraSeal RC Sealer, Meta Biomed, South Korea) (Figure 3f).

At 1‐week review, the patient was asymptomatic with no tenderness to percussion or palpation in either tooth. The orthodontic relapse of midline diastema was discussed with the patient, and the patient agreed to full coverage crown. At 6‐ and 12‐month follow‐up, both incisors remained functional and asymptomatic (Figure 3g–i). The patient was comfortable during the treatment and had no adverse events. Moreover, he was satisfied with the treatment outcome.

## 3. Discussion

One measure of pulpal health is pulpal blood flow. Teeth sensitivity and pulpal sensory responses may decline as a result of temporary ischemia or reduced pulpal blood flow. [6]

In teeth with complete apical development, orthodontic tooth movement may result in inflammatory and/or degenerative reactions in the dental pulp. The neurovascular system, where the release of certain neurotransmitters (neuropeptides) can affect both blood flow and cellular metabolism, is the main area where tooth movement affects pulp [7], as evidence has shown an increase in pulp canal obliterations after orthodontic treatment (Table 1).

**Table 1:** Pulpal calcific changes following orthodontic treatment.

Author (year of publication)	Type of study	Relevant findings
Afsari et al. (2021) [8]	Observational	There was a notable rise in pulp stones in the orthodontic group′s first molars (*p* = 0.023) and second molars (*p* = 0.002). None of the teeth in the nonorthodontic group had a noticeably higher pulp stone count.
Ertas et al. (2017) [9]	Observational	Prior to orthodontic therapy, there were 257 pulp stones (3% of teeth), and following treatment, there were 437 (5.2% of teeth). The incidence of pulp stones on radiographs taken before and after therapy increased significantly (2.2%) (*p* < 0.001).
Han et al. (2013) [10]	Clinical laboratory	Week 8: The severe force group had multiple pulp stones, whereas the moderate force group had none. Week 12: The coronal and root pulps had several pulp stones and calcified lines surrounding the dentin; the moderate force group did not have any pulp stones in the root pulp.
Singh et al. (2018) [11]	Observational	Of the 200 patients, 23 (11.5%) had pulp stones prior to starting orthodontic treatment, and 31 (15.5%) had them afterward. The 4% rise in instances was statistically significant (*p* < 0.05).
Korkmaz et al. (2019) [5]	Observational	36 individuals had pulp stones prior to orthodontic therapy; 160 patients had pulp stones following orthodontic treatment. Following therapy, there was a significant increase in pulp stones (*p* < 0.05).

In all human instances with higher orthodontic forces, Hamilton and Gutmann (1936, 1937) demonstrated some indications of severe pulpal degeneration. According to his research, the primary cause of pulpal degeneration is the absence of collateral circulation to the pulp during tooth movement. In order to lessen harm to the tooth tissues and give time for potential repair, he advised using light intermittent forces [7].

Orthodontic forces are applied to multiple teeth, yet obliteration is often localized. A balanced interpretation incorporates several localized and individual factors [12]:

• Localized vascular compromise: Intrusion or specific force vectors may transiently compromise apical blood flow in a single tooth, triggering hard‐tissue deposition responses unique to that tooth′s vascular anatomy.
• Force magnitude and direction: Variations in anchorage, bracket slot engagement, and archwire mechanics can result in higher or more sustained forces on individual teeth; intrusion and tipping movements are particularly associated with pulpal changes.
• Root morphology and canal anatomy: Teeth with narrower canals or pre‐existing sclerotic changes may be more prone to obliteration under similar force systems.
• Individual biological variability: Genetic and cellular differences in odontoblast activity and pulp healing capacity can lead to markedly different responses among teeth within the same arch.

From an endodontist′s perspective, obliterated teeth present two main challenges: unreliable sensibility testing and high risk of iatrogenic damage during conventional access. Reviews emphasize that many obliterated teeth remain vital despite negative responses to thermal and electric tests, so diagnosis must integrate symptoms, radiographic appearance and, increasingly, CBCT imaging to visualize residual canal pathways and assess cortical bone thickness. Conventional freehand access—using magnification, multiple exploratory radiographs, and small hand files—remains feasible in mildly to moderately calcified canals but is associated with increased incidence of perforation, excessive removal of pericervical dentin, and procedural errors in severely obliterated roots. GE represents a new approach to reduce issues including technical malfunctions, random alterations in root canal geometry, and significant dentine loss that might weaken teeth or result in root perforations. [13] The customized 3D printed guide that was fabricated using CBCT images and digital planning software aided in locating the root canal space. The canal was successfully navigated with the help of the EndoGuide bur, which was oriented using the guide. EndoGuide bur helped in efficient removal of calcification, precise navigation, and ultimately improved treatment outcome. GE seems to be an accurate and reliable method for treating teeth with pulpal canal calcification. [14, 15]

## 4. Limitations

Despite its advantages, GE is not free from complications. It can cause intraoperative deviation of the drill path resulting in labial perforation due to limited sleeve length, inappropriate bur diameter and stiffness, and possible guide misfit. And due to high cost and limited availability of CBCT, intraoral scanner, and 3D printer in regular practice, it becomes difficult for regular practitioners to incorporate it in every calcified case [16] Moreover, a previous undocumented traumatic event cannot be completely excluded as a contributing factor to the observed canal obliteration.

To the best of our knowledge, this is the only case report where the management of orthodontic‐induced canal calcification has been documented and managed.

## 5. Conclusion

This case demonstrates the importance of interdisciplinary treatment planning. The successful management of obliteration arising from orthodontic tooth movement, combined with effective closure of diastema, reveals its significance. However, long‐term follow‐up remains essential to assess treatment stability.

## Author Contributions

Ihtisam Alam: investigation, data curation, and writing—original draft. Aravind R. Kudva: conceptualization, supervision, and writing—review and editing. S. Vidhyadhara Shetty: supervision. Aboobacker Sidheeque: writing—review and editing.

## Funding

No funding was received for this manuscript.

## Disclosure

All authors have read and approved the final version of the manuscript. As the corresponding author and manuscript guarantor, I had full access to all the data in this study and take complete responsibility for the integrity of the data and the accuracy of the data analysis.

## Ethics Statement

Ethical approval was not required because this manuscript reports a single anonymized clinical case. Written informed consent for treatment and publication was obtained from the patient.

## Consent

This reporting case follows the CARE guidelines, and written informed consent was obtained from the patient. No data on the paper reveals the patient′s identity.

## Conflicts of Interest

The authors declare no conflicts of interest.

## Data Availability

The data that support the findings of this study are available on request from the corresponding author. The data are not publicly available due to privacy or ethical restrictions.
